# A Brief Observation to Screen Autism in Toddlers and Predict Developmental Trajectory

**DOI:** 10.1016/j.jpedcp.2025.200176

**Published:** 2025-09-01

**Authors:** Fiona Journal, Thibaut Chataing, Michel Godel, Nada Kojovic, Kenza Latrèche, Maude Schneider, Marie Schaer

**Affiliations:** 1 Faculty of Medicine, Department of Psychiatry, University of Geneva, Geneva, Switzerland; 2 Faculty of Psychology and Science of Education (FAPSE), University of Geneva, Geneva, Switzerland; 3 Division of Adult Psychiatry, Department of Psychiatry, University Hospitals of Geneva, Geneva, Switzerland

**Keywords:** autism, decision tree, early diagnosis, machine learning, predictors, screening tool

## Abstract

**Objective:**

Autism spectrum disorder (ASD) affects approximately 1 in 31 children. Early diagnosis is crucial for optimizing outcomes through early interventions, and primary care settings need efficient tools to identify children presenting autistic features. This study explores the potential of early socio-communicative behaviors, measured by the Early Social Communication Scales, to screen for ASD in children younger than 3 years old, and predict their future cognitive development using machine learning models.

**Study design:**

This study analyzed longitudinal data from 113 children with ASD and 59 with typical development (TD), aged from 1 to 3 at baseline. Twenty-three ESCS variables were used to screen for ASD and predict cognitive development. The C5.0 decision tree algorithm was used to classify ASD vs TD, while linear regression and K-means clustering identified cognitive development patterns among autistic children. K-fold cross-validation, permutation testing, and undersampling were used for validation.

**Results:**

We distinguished between ASD and TD children with 95% accuracy, 96% sensitivity and 92% specificity. Nine behaviors contributed to distinguish ASD from TD. Behaviors that contributed most are the child's ability to initiate a turn taking and to point at desired objects. A separate model stratified children into groups with different cognitive outcome with 97% accuracy. Behavioral requests variables contributed in distinguishing extreme cognitive trajectories in autistic children.

**Conclusions:**

We provide an original decision-algorithm focusing on early socio-communicative behaviors to guide pediatricians through autism screening and cognitive development prediction.

## Highlights

• A decision-tree algorithm to improve early autism diagnosis and predicts developmental trajectory.
• Enhances diagnostic accuracy and cognitive development prediction in a short assessment time.

Autism spectrum disorder (ASD) affects approximately 1 in 31 children in the USA.^1^ Despite advances in understanding the genetic and neural bases of ASD, diagnosis still relies on time-consuming behavioral assessments. Such evaluations are done by trained professionals, leading to long waiting times, significant costs, and delayed access to appropriate care.^2^^,^^3^ However, early diagnosis is crucial for implementing interventions that enhance developmental outcomes and later quality of life for autistic children.^4^, ^5^, ^6^, ^7^, ^8^ In this regard, effective and easy-to-administer screening tools in primary care settings are essential, enabling pediatricians to identify children displaying features of ASD earlier, facilitating access to diagnostic and interventions services. Recently, there has been increasing interest in machine learning to assist in diagnosing ASD. Machine learning has been applied in various datasets (eg, genetic,^9^^,^^10^ neuroimaging,^11^, ^12^, ^13^, ^14^, ^15^ and environmental data^16^^,^^17^) to capture the complexity of ASD,^18^, ^19^, ^20^, ^21^, ^22^, ^23^ enhancing diagnostic precision through identifying key features for diagnosis,^24^, ^25^, ^26^, ^27^, ^28^ and response to intervention.^29^ To track autism markers, these studies have often relied on parent-reported questionnaires^30^, ^31^, ^32^, ^33^, ^34^, ^35^, ^36^, ^37^, ^38^ that could be subject to completion bias^39^ or standardized assessments like the Autism Diagnostic Observation Schedule (ADOS)^38^^,^^40^, ^41^, ^42^, ^43^ and the Autism Diagnostic Interview^31^^,^^38^ that are difficult to implement in a primary care setting because of their duration and the experience they require.

In this study, we propose an alternative approach by exploring a targeted subset of socio-communicative behaviors from the Early Social Communication Scales (ESCS)^44^ as potential ASD markers. The ESCS is a brief (15–25 minute), semi-structured assessment tool that directly measures socio-communicative skills in children under 3 years of age. Early social-skills are pivotal to cognitive outcomes in ASD, and are the primary target of many early intervention models aimed at improving long-term cognitive functioning.^45^^,^^46^ Although the ESCS is widely used in research to assess early social-communicative behaviors, it has rarely been applied in screening contexts and, to our knowledge, has never been integrated into a data-driven model to classify ASD or predict cognitive outcomes.

One of our previous studies confirmed a link between lower levels of early socio-communicative skills and poorer cognitive development in children with ASD.^47^ In the field of machine learning, one study examined the prediction of intellectual disability in ASD, based on questionnaires completed retrospectively by parents and found that the strongest predictors of intellectual impairment were parents-reported measures of language delay, general cognitive delay and younger age at diagnosis.^48^ However, machine learning studies have yet to consider direct measures of early social behaviors to predict cognitive development in ASD. In this work, decision tree algorithms were selected for their ability to model complex relationships between variables while remaining highly interpretable and clinically intuitive. Unlike many machine learning models, decision trees provide transparent, step-by-step decision rules that can be easily followed by practitioners, making them especially well-suited for early screening and developmental monitoring in primary care.

Here, we aim to provide practical insights for primary care practitioners by identifying which early social behaviors could serve as screening tools for autism and cognitive outcome prediction by age 5. Our objectives are: (1) to build a robust decision tree using a clinically useful subset of ESCS items to classify children under 3 years of age as having or not having a diagnosis of ASD and (2) to examine whether early socio-communicative skills, observed through simple, easily assessable activities, can predict cognitive outcomes in autistic children up to age 5 using a decision tree model.

## Methods

### Study Design and Setting

This prospective study analyzed data from the longitudinal Geneva Autism Cohort,^49^ established in 2012. Children were enrolled through local clinical centers, parent associations, or word-of-mouth. The research protocol received approval from the University of Geneva's Cantonal Research Ethics Committee (CCER-Commission cantonale d’éthique de la recherche) and written informed consent was obtained from all parents.

Our sample included 113 autistic children and 59 typically developing (TD) children who completed a valid ESCS evaluation between 1 and 3 years of age at baseline. From the ASD sample, 111 had multiple evaluations, yielding 480 timepoints. ASD diagnosis was confirmed by experienced clinicians based on Diagnostic and Statistical Manual of Mental Disorders, Fifth Edition criteria,^50^ using a best-estimate clinical judgment that integrated information from the ADOS^51^^,^^52^ and the ADI-Revised. TD children were also assessed using the ADOS to ensure the absence of ASD symptoms. The ADOS-G^53^ version was used before 2016 and the ADOS-2^52^ was used thereafter. To ensure consistency, all ADOS-G evaluations in our sample were rescored using the ADOS-2 algorithm. ADOS modules were selected based on age and language level, and the calibrated severity score^51^ was use to allow comparison across modules. Description of both groups is given in Table. As shown, the ASD and TD groups differ in male-to-female ratio, with a higher proportion of boys in the ASD group. This reflects the known male predominance in autism prevalence.^1^ Although sex was not included as a predictor in the decision-tree models, this imbalance may influence certain behavioral patterns and should be considered when interpreting the findings.

**Table I:** Group comparison of developmental variables between children with autism spectrum disorder (ASD) and typically developing (TD) peers at the first timepoint

Variable	ASD	TD	T	Df	*P* value	Cohen's d	95% CI (mean diff)
Age							
Mean	**2.35** (n = 113)	**2.01** (n = 59)	4.344	171	**<.001**	0.70	[0.17; 0.51]
Min	1.04	1.03					
Max	3.00	2.98					
SD	0.45	0.54					
Developmental quotient							
Mean	**67.93** (MSEL: 97/PEP: 16) 32 intellectual disability	**112.20** (MSEL: 44/PEP: 15) 0 intellectual disability	−16.965	150.25	**<.001**	−2.67	[-50.42; −39.26]
Min	20.48	87.23					
Max	144.79	160.13					
SD	19.40	14.39					
ADOS total severity score							
Mean	**7.56** (n = 94)	**1.04** (n = 50)	31.542	96.44	**<.001**	4.97	[6.93; 7.26]
Min	2	1					
Max	10	2					
SD	1.98	0.20					

Variable	ASD	TD	Z	*P*-value	Φ
Gender	22 female (19.3%)	24 female (40.7%)	3.017	**0.003**	0.23

Statistical comparisons were conducted using independent-samples t-tests and proportions tests.

Significant differences are highlighted in bold.

### Evaluations

#### ESCS

The ESCS, designed as a practical research instrument, as well as a clinical tool, assesses early socio-communicative development in young children through semi-structured activities.^44^ The scoring emphasizes frequency data, rather than ordinal. Indeed, all the child's behaviors are counted and divided according to their purpose in interaction: a behavior to share interest (Joint Attention domain); to request (Behavioral requests domain) or to initiate and maintain an interaction (Social interaction domain). These behaviors include nonverbal means of communication (eg, eye contact, pointing, coordination of several gestures) and are sub-divided into high- and low-level depending on their complexity (Supplementary Table 2). Children were included if they were regularly exposed to either French or English. The ESCS tasks primarily elicit nonverbal social-communicative behaviors (eg, gaze, gestures, pointing), and verbal production was not included in the variables used for classification. Assessments were administered by trained clinicians who received standardized instruction in ESCS administration and scoring, with regular supervision to ensure consistency. The ESCS's validity and reliability are well-established.^54^, ^55^, ^56^, ^57^

### Cognitive Development

Two autistic children were excluded from the cognitive analysis as they only had a single timepoint. Overall developmental age was calculated based on standardized scores in the various domains for each of the evaluations (Mullen Scales of Early Learning, Psychoeducational Profile, and Wechsler Preschool and Primary Scale of Intelligence). Different cognitive assessments were used depending on the child's age and level of development. The *MSEL (Mullen Scales of Early Learning)*^58^ is a standardized tool for children aged birth to 68 months, assessing gross and fine motor skills, expressive and receptive language and visual reception. *The PEP (Psychoeducational Profile - 3rd ed.)*^59^ is a structured tool for children aged 6 months to 7 years, evaluating gross and fine motor skills, expressive and receptive language, imitation and global cognitive abilities. The *WPPSI-IV (Wechsler Preschool and Primary Scale of Intelligence)*^60^ is a standardized test assessing the IQ for children aged 2.5 to 7.5 years, measuring verbal reasoning, working memory, perceptual reasoning, and processing speed.

### Statistical Analyses

#### General Algorithm: Decision Tree Analysis

Decision tree is the most fundamental and widely used classification method in the machine learning field. The classifier is constructed using the C5.0^61^ package implemented in R^62^ (R Core Team, R Foundation for Statistical Computing, Vienna, Austria, version 2023.12.1). C5.0 uses gain ratio as splitting criteria, at each node, the attribute offering the best information gain is chosen to divide the data, and the division ceases when all examples in a node belong to the same class. In this study, the Winnow option of C5.0 method is used, it examines the usefulness of the predictors estimating the error rate without each predictor and comparing it to the error rate when all the predictors are used. If the error rate improves without the predictor, it is then excluded from the model-building process.

### Model Evaluation

As our sample size does not allow splitting into a training and test sample (According to,^63^ sample sizes from 120 reduced discrepancy in accuracy), we validated the classification model created with the C5.0 algorithm using three approaches: K fold-Validation, Permutation testing and Undersampling. The combination of three approaches enables us to check the validity of the decision tree on part of our data (K-fold validation), to ensure that the decision tree reflects the meaning of the data and not random chance (Permutation Testing) and to compensate for the imbalance in numbers between our groups by checking that the results do not favor the largest class (Undersampling). Details of these validations are available in Supplementary Analysis 1). Confidence intervals for accuracy, sensitivity, and specificity were calculated using the Wilson score method for proportions.

### Variables Importance

Feature importance analysis (function *C5imp* in R) quantifies the relationship between each predictor and the target variable, expressed through an importance coefficient. Predictors involved in the first split inherently receive an importance score of 100%, as they directly influence the initial partitioning of the data. The underlying concept of feature importance measurement involves assessing how much the model's performance degrades when the values of a specific feature are randomly shuffled or permuted, while all other features remain unchanged. This analysis enables us to identify which behaviors are most decisive in the screening for ASD.

### Clusters on Cognitive Development

To predict the cognitive development of autistic children at 5 years old based on their early socio communicative skills, children assessed at least twice in our protocol were retained. For each of the 111 children, a linear regression analysis was performed on developmental age scores in Matlab.^64^ The slope coefficient was then used to construct different groups of autistic children based on their cognitive evolution until 5 years old with a K-means clustering approach on IBM SPSS Statistics (IBM Corp, Armonk, New York, version 29.0.0.0).^65^ Based on a previous study of our group,^46^ we hypothesized to identify 3 groups of children. Clusters were defined in a data-driven manner using the individual slope of cognitive progression over time. No fixed cutoff score was applied to distinguish higher or lower cognitive functioning; instead, the K-means algorithm grouped children based on shared developmental trajectories. To check the validity of clusters, the silhouette coefficient of coherence and separation was used, which ranges from −1 to 1. Comparison of the three clusters on Chronological Age, Developmental Age and ADOS severity scores at baseline were done on SPSS^65^ using ANOVA. Post hoc analyses were done using T-tests adjusted using Bonferroni Correction for all variables, except for ADOS severity scores that are not normally distributed, compared using a Kruskal–Wallis’ test corrected by Dunn's Test.

## Results

### A Decision Tree for Autism Screening in Toddlers

**Figure 1:**
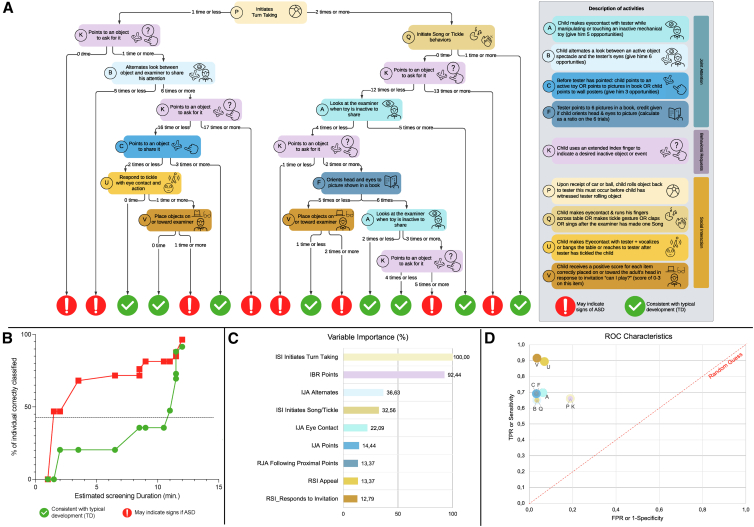
Decision tree model for early autism screening based on ESCS behaviors. **A,** Screening decision tree with behavior thresholds guiding classification. ESCS domains are color-coded: Joint Attention (*blue*), Behavioral Requests (*purple*), and Social Interaction (*ochre*). Variables are labelled according to Supplementary Table 2. **B,** Estimated duration of the tool, showing cumulative classification accuracy as a function of the time needed to complete each activity. **C,** Feature importance scores (%) for variables contributing to the screening model. **D,** ROC characteristics plot showing model accuracy for autism screening classification (TPR = true positive rate; FPR = false positive rate).

The performance indicators were computed by treating ASD as positive and TD as negative cases. The diagnostic classification decision tree achieves an accuracy of 95% (95% CI [89.4%, 97.0%]) (Figure 1A), a sensitivity of 96% ([90.1%, 98.3%]) and a specificity of 92% ([81.3%, 96.5%]) (Details and validation in Supplementary Figure 1 and -Analysis 2). Rather than relying on the full ESCS, the final decision tree is based on a subset of 9 variables that proved most effective in distinguishing ASD cases. These variables correspond to socio-communicative behaviors that are typically elicited through simple and intuitive activities, such as rolling a ball, showing a book, or pointing to request an object, which require minimal materials and can be easily implemented in clinical interactions. To better reflect the duration of the evaluation, the percentages of detection expressed as a function of the estimated observation time are illustrated in Figure 1B (see Supplementary Table 1 for details of the estimation).

### Variables Importance

Importance of the variables contained in the diagnostic classification tree is reported in Figure 1C. To more accurately assess the contribution of each variable to the final diagnostic classification tree, we calculated the performance metrics of dummy trees in which the variables were added one by one (Figure 1D).

The two first variables (K and P) effectively classify 53 of the ASD children from TD peers (Supplementary Figure 1). Another group of variables (A, B, C, F, and Q) increase the specificity of the model, and a third group of variables (U and V) increase its sensitivity.

### Clusters Based on Cognitive Development

**Figure 2:**
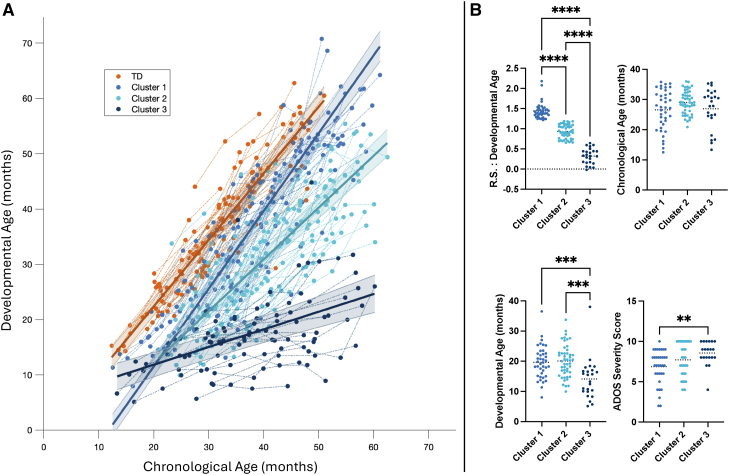
Cognitive trajectories and cluster characteristics among ASD children. **A,** Longitudinal developmental trajectories based on cluster membership, modelled using mixed-effects regression.^67^^,^^68^ Individual timepoints are connected by thin lines; group-level predictions are shown with solid lines and 95% confidence intervals by coloured bands. ***B*,** Cluster comparisons at baseline, showing developmental and chronological age, regression slopes (R.S.) of developmental age and ADOS Severity Scores. T-tests (with Bonferroni correction) and Kruskal-Wallis tests (with Dunn's post hoc correction) were used. ∗∗ = P < .001; ∗ = P < .05.”

To explore whether early socio-communicative skills could predict cognitive progress in children with ASD, the first step consisted of grouping autistic children into clusters demonstrating similar patterns of cognitive evolution, to next apply the same decision tree analysis as used for autism screening, this time using cluster membership (describing different cognitive development) as outcome variable. These clusters reflect data-driven profiles of cognitive development derived from individual regression slopes (Figure 2A).

The cluster analysis yielded 3 clusters (average silhouette coefficient = 0.7), referred to as Cluster 1 (n = 39), Cluster 2 (n = 48) and Cluster 3 (n = 24). These clusters differed significantly on developmental age at the first timepoint (ANOVA: F(2,108) = 9.785, *P* < .001, η^2^ = 0.15), slope of developmental age over time (ANOVA: F(2,108) = 270.44, *P* < .001, η^2^ = 0.83) and ADOS Severity Score (χ2(2) = 9.105, *P* = .011) but not on chronological age (ANOVA: F(2,108) = 2.410, *P* = .095). Post-hoc comparisons corrected by Bonferroni's procedure (Figure 2B) revealed that Cluster 3 showed weaker cognitive skills at baseline compared to Cluster 1 (Mean difference = 5.53, *P* = .001, Cohen's d = 0.90, 95% CI [2.63-8.43]) and Cluster 2 (mean difference = 5.98, *P* < .001, d = 1.00, 95% CI [3.26-8.70]). Cluster 1 showed a faster cognitive development over time compared to Cluster 3 (mean difference = 1.12, *P* < .001, Cohen's *d* = 5.78, 95% CI [1.01-1.23]) and compared to Cluster 2 (mean difference = 0.51, *P* < .001, *d* = 2.77, 95% CI [0.42-0.60]). Cluster 2 also progressed more rapidly than Cluster 3 (mean difference = 0.61, *P* < .001, *d* = 3.33, 95% CI [0.50-0.72]). Cluster 3 also showed greater ADOS Severity scores than Cluster 1 (Kruskall-Wallis: Mean Rank Difference = −22.55, *P* = .009, r = 0.36). Together, these results indicate that Cluster 1 included children with higher baseline cognitive scores and faster cognitive development, Cluster 2 showed intermediate profiles both at baseline and over time, and Cluster 3 was characterized by the lowest initial developmental scores, the slowest cognitive progress, and greater autism symptom severity.

### Decision-Tree-Based Screening Tools for the Cognitive Development of ASD Children

To examine whether some early socio-communicative skills could predict the cognitive evolution of autistic children at 5 years old, we conducted the same decision tree analysis as used for autism screening, this time using the three cognitive trajectory clusters as a classification variable. However, the model was not validated (cross-validated Kappa = 0.654 and no performance advantage over permuted models; see Supplementary Analysis 3). We therefore focus on the 2 most divergent cognitive outcomes: Cluster 1 (who reached typical developmental levels by age 5) and Cluster 3 (children with minimal cognitive progress). Cluster 2, with more heterogeneous developmental trajectories, was excluded from the final classification model.

**Figure 3:**
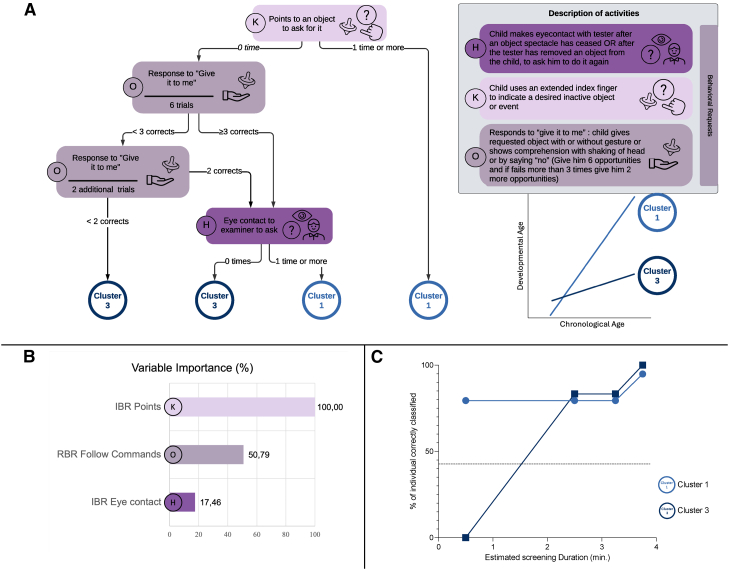
Decision tree model for predicting developmental trajectory in toddlers with ASD. **A,** Classification tree with behavior thresholds guiding prediction of developmental trajectory. All behaviors are from the Behavioral Requests domain of the ESCS (*purple*). Variables are labelled according to Supplementary Table 2. **B,** Feature importance scores (%) for variables included in the developmental trajectory model. **C,** Estimated duration of the tool, showing cumulative classification accuracy for developmental trajectory prediction as a function of the time needed to complete each activity.”

This resulting binary decision tree achieved a classification accuracy of 97% (Figure 3A and Supplementary Figure 2), with 95% sensitivity and 100% specificity (see Supplementary Analysis 4 for validation details). Three ESCS variables were retained as key predictors of cognitive outcome: (1) whether the child looks at the examiner to request an object (H), (2) points to request an object (K), and (3) responds to “give it to me” prompts (O).

To explore whether the decision tree could generalize beyond the extreme cognitive profiles, we applied it post-hoc to the intermediate group (Cluster 2). The model classified 35 children as “Cluster 1-like” and 13 as “Cluster 3-like.” Those classified as Cluster 1-like showed significantly higher cognitive scores at both baseline and follow-up, despite similar ages and rates of cognitive growth (see Supplementary Analysis 5). These results suggest that early socio-communicative behaviors may help differentiate developmental outcomes even within profiles that do not follow extreme cognitive trajectories.

### Variables Importance

Variable importance analysis is applied to better understand the specific characteristics of better or worse cognitive evolution in ASD children; results are shown in Figure 3B.

## Discussion

This study tested whether a few easily observable behaviors can distinguish children with and without autism or help predict future cognitive outcomes in autistic children. Using ESCS evaluation and decision-tree analysis, it offers practical tools for pediatricians to enhance routine screenings.

### A Decision Tree for Autism Screening in Toddlers

The classification model identified socio-communicative patterns that distinguished individuals with ASD from those without, achieving over 85% accuracy after K-fold cross-validation and controlling for unbalanced data. The proposed screening tool (Figure 1A.) is based on behaviors displayed by the child, offering good granularity in their evaluation but easier to implement for nonspecialist clinicians and takes less time than gold-standard diagnosis assessments.

Although variable importance rankings depend on the model and dataset, several behaviors emerged as especially informative for early ASD screening. Two variables: initiating ball passes (P) and pointing to request objects or actions (K), were sufficient to correctly classify 53 of the 113 autistic children. These behaviors reflect distinct communicative functions^66^: social initiation (throwing a ball) and instrumental request (pointing to an object). Observing just these two behaviors can lead to the identification of 47% of the autistic children in under 2 minutes (Figure 1B), making them especially useful for rapid screening.

A second group of variables, including behaviors related to joint-attention skills^67^ (B, A, C, F) and initiating social interaction (Q), improved the model's specificity. These initiation-based skills, particularly those with a social function, are consistent with established early markers of autism.^68^, ^69^, ^70^, ^71^ and helped identify up to 81% of autistic children and 47% of typically developing peers within 11 minutes.

The model also considers combinations of behaviors that, while rare, are clinically meaningful. For instance, the co-occurrence of very frequent alternated gazes between examiner and object (B) and pointing to ask for objects (K) flagged a very small subgroup of autistic children (0.05%) with atypical behavior profiles. Despite their specificity, these profiles were reliably distinguished from typically developing peers when the full tree was applied, achieving 95% accuracy within a 12-minute observation window.

Finally, a third set of variables involved responses to social prompts (V). These were informative primarily when paired with low frequencies of other key behaviors (A, K, F), suggesting that the absence of certain foundational skills may influence how social responses are interpreted by the model.

Together, these results support a structured, step-by-step interpretation of the decision tree. We recommend that clinicians pay particular attention to a child's ability to initiate both social and instrumental communication, as these behaviors were most predictive of ASD in toddlers and are easily observable in clinical settings.

The second-level screening solutions currently available are STAT,^72^ a video device from,^73^ BOSA,^74^ RITA-T^75^ and a screening app from,^76^ taking between 10 and 20 minutes and having a sensitivity between 86 and 100% and a specificity between 67 and 95% in the same age range. Our tool falls within a similar time range (12 minutes) and offers strong performance, with a sensitivity of 96% and specificity of 92%. Moreover, its step-by-step structure makes it a dynamic process as shown in Figure 1B, observing just the first two behaviors (which takes approximately 1.5 minutes) is sufficient to identify 47% of autistic children. This flexibility allows clinicians to adapt the tool to the time available, making it a promising and relatively easy-to-use second-level screening option for autism detection in primary care.

### Decision-Tree-Based Screening Tools for the Cognitive Development of ASD Children

The classification model identified socio-communicative patterns stratifying with >90% accuracy, ASD children who later reach typically developing peers’ cognitive level, and autistic children who show poorer cognitive evolution. It should be noted that a model attempting to classify all ASD children into 3 cognitive trajectories showed lower performance (Supplementary Analysis 2). Although Cluster 2 was excluded from model training to enhance classification accuracy, its post hoc classification demonstrated that early socio-communicative markers may also differentiate children with intermediate developmental trajectories. This exploratory analysis supports the potential generalizability of the tool, though validation in independent samples remains necessary (Supplementary Analysis 5). These results suggest that ESCS variables can capture clinically relevant developmental profiles beyond binary extremes, using a brief observation of less than 4 minutes (Figure 3C)

One previous study^48^ looked at the prediction of intellectual disability in ASD using machine learning methods, based on questionnaires filled-in retrospectively by parents about their child and revealed that language difficulties, cognitive delay and a younger age at diagnosis, reported by parents, are shown to be the most predictive variables of subsequent intellectual disability. To our knowledge, no studies have considered early social behaviors used by children to predict their cognitive development using machine learning methods. Considering the small sample size of this study, testing these models on larger samples is necessary.

In this study, we identified 3 behaviors: looking at the examiner to request an object (H), pointing to ask for it (K), and responding to a “give it to me” prompt (O), as key predictors of cognitive development, all belonging to the behavioral request domain. These early-emerging skills, typically acquired between 2 and 7 months in typical development, serve an instrumental communication function that allows the child to express basic needs.^57^ Although considered foundational, these behaviors appear to support the emergence of more complex social skills and are therefore critical for cognitive growth. While all socio-communicative skills are important in early intervention,^71^^,^^77^^,^^78^ focusing on behavioral requests may help identify children at risk of slower developmental progress.^79^ As such, this decision-tree tool could complement diagnostic screening by offering a quick way to estimate developmental prognosis and tailor early support accordingly.

Our findings contribute to the clinical goal of early ASD screening and individualized intervention by proposing a brief, interpretable tool suitable for use in primary care. We selected a decision tree approach (C5.0) for its strong alignment with clinical reasoning: it automatically models complex relationships while generating intuitive, rule-based outputs based on directly observable behaviors. This structure supports rapid and transparent decision-making, consistent with the workflow of pediatric practice. Rather than relying on the full ESCS protocol, the proposed tool uses a simplified set of nine socio-communicative behaviors adapted for independent use. These interactions are short, naturalistic, and based on common early developmental scenarios (eg, pointing to a picture book, turn-taking with a ball, requesting toy activation). They require minimal materials and are likely to be intuitive for clinicians accustomed to interacting with young children. Although no formal training protocol was evaluated, the publicly available ESCS manual provides clear guidelines for each activity44 (Specific Task Administration Guidelines). Clinicians with prior experience in child interaction could likely become autonomous in administering the screening tool after approximately 2 hours of familiarization with the manual and practice of the tasks. In future applications, an app-based interface could support implementation by guiding clinicians through each step and providing real-time instructions. A limitation of this study is that the model was developed and validated using the same dataset. Although we applied pruning, automatic feature selection, undersampling, and cross-validation to minimize overfitting, some optimism in performance may remain. Future studies should replicate these findings in larger, independent samples and evaluate how cultural, linguistic, and clinical diversity may affect implementation and generalizability. While this tool provides a rapid, structured screening approach, it is not intended to replace comprehensive clinical evaluations. As with all machine learning–based tools, its outputs must be interpreted within the broader clinical context, and decisions regarding diagnosis or intervention should always involve multidisciplinary clinical judgment.

This study highlights that a small set of early, nonverbal socio-communicative behaviors can accurately predict both autism diagnosis and later developmental trajectory through an interpretable decision tree model. The approach aligns with pediatric reasoning and emphasizes clinically observable behaviors, supporting its potential use in real-world settings. However, further validation in independent and more diverse samples is necessary to confirm generalizability and clinical impact.

## Data Statement

Data sharing statement available at www.jpeds.com.

## Declaration of Generative AI and AI-Assisted Technologies in the Writing Process

During the preparation of this work, the authors used Chat GPT in order to improve language and readability. After using this tool, the authors reviewed and edited the content as needed and take full responsibility for the content of the publication.

## CRediT authorship contribution statement

**Fiona Journal:** Writing – review & editing, Writing – original draft, Visualization, Methodology, Formal analysis, Conceptualization. **Thibaut Chataing:** Writing – review & editing, Conceptualization. **Michel Godel:** Writing – review & editing, Conceptualization. **Nada Kojovic:** Writing – review & editing, Investigation. **Kenza Latrèche:** Writing – review & editing, Conceptualization. **Maude Schneider:** Writing – review & editing, Supervision, Conceptualization. **Marie Schaer:** Writing – review & editing, Validation, Supervision, Project administration, Investigation, Funding acquisition, Data curation, Conceptualization.

## Declaration of Competing Interest

This work was supported by the Swiss National Center of Competence for Research (NCCR) Synapsy (Grant No. 51NF40–185897), by grants from the Swiss National Foundation for Scientific Research (Grant Nos. #163859, #190084, #202235, #212653 to M.S.), by the Fondation Privée des Hôpitaux Universitaires de Genève (https://www.fondationhug.org), and by the Fondation Pôle Autisme (https://www.pole-autisme.ch). The funders were not involved in this study and had no role other than to provide financial support. Authors have no conflicts of interest to disclose.

## Supplementary Data

Supplementary Materials

Data Statement
