# Supplementary material for: A Brief Observation to Screen Autism in Toddlers and Predict Developmental Trajectory
Source: J Pediatr Clin Pract. 2025 Sep 1;18:200176. doi: 10.1016/j.jpedcp.2025.200176 (PMC12766091; doi:10.1016/j.jpedcp.2025.200176)
Supplement: Supplementary Materials [file mmc1.docx]

**Supplementary Material**

|  | **DOMAIN** | **LEVEL** | **CODE** | **LETTER** | **TASKS** | **DESCRIPTION** |
| --- | --- | --- | --- | --- | --- | --- |
| Joint Attention | Initiation of Joint attention | Lower | **IJA_EyeContact** | **A** | Activation of mechanical toy | Child makes eye contact with tester while manipulating or touching an inactive mechanical toy |
|  |  | Lower | **IJA_Alternates** | **B** | Activation of mechanical toy | Child alternates a look between an active object spectacle and the tester’s eyes |
|  |  | Higher | **IJA_Points** | **C** | Activation of mechanical toy  + Book | Before tester has pointed: child points to an active toy OR points to pictures in book OR child points to wall posters |
|  |  | Higher | **IJA_Points+EyeContact** | **D** | Activation of mechanical toy | IJA_Points occuring with eye contact |
|  |  | Higher | **IJA_Show** | **E** | Activation of mechanical toy | Child raises a toy upward toward tester’s face to show him but not giving him |
|  | Response to Joint Attention | Lower | **RJA_FollowingProximalPoints** | **F** | Book | Tester points to 6 pictures in a book, credit given if child orients head & eyes to picture (calculate as a ratio on the 6 trials) |
|  |  | Higher | **RJA_FollowingLineOfRegard** | **G** | Look at poster on the wall | Tester point to poster on the wall and child receives credit if they turn eyes or head sufficiently to indicate they are looking in correct direction (calculate as a ratio on the 8 trials) |
| Behavioral Requests | Initiation of Behavioral Requests | Lower | **IBR_EyeContact** | **H** | Activation of mechanical toy | Child makes eye contact with tester after an object spectacle has ceased OR after the tester has removed an object from the child, to ask him to do it again |
|  |  | Lower | **IBR_Reach** | **I** | Activation of mechanical toy | Child extends arm toward an out of reach toy |
|  |  | Lower | **IBR_Appeal** | **J** | Activation of mechanical toy | Child combines IBR_Eyecontact with IBR_Reach |
|  |  | Higher | **IBR_Points** | **K** | Activation of mechanical toy | Child uses an extended index finger to indicate a desired inactive object or event |
|  |  | Higher | **IBR_Point+EyeContact** | **L** | Activation of mechanical toy | Child uses pointing and eye contact to indicate a desired inactive object or event |
|  |  | Higher | **IBR_Give** | **M** | Activation of mechanical toy,  Toy in a jar | Child pushes object toward tester OR holds an object out toward tester (typically towards tester’s hands or body) |
|  |  | Higher | **IBR_Give+EyeContact** | **N** | Activation of mechanical toy,  Toy in a jar | Child pushes object toward tester OR holds an object out toward tester with eye contact |
|  | Response to Behavioral Requests | None | **RBR_Follow Commands** | **O** | Activation of mechanical toy,  Toy in a jar | Responds to “give it to me”: child gives requested object with or without gesture or shows comprehension with shaking of head or by saying “no” |
| Social Interaction | Initiation of Social Interaction | None | **ISI_Initiates_TurnTaking** | **P** | Turn taking | Upon receipt of car or ball, child rolls object back to tester this must occur before child has witnessed tester rolling object |
|  |  | None | **ISI_Initiates_Song/Tickle** | **Q** | Tickle and song | Child makes eye contact & runs his fingers across table OR makes tickle gesture OR claps OR sings after the 1st SONG/TICKLE task has been presented |
|  |  | None | **ISI_Tease** | **R** | - | Child engages in a prohibited act while displaying positive affect toward tester (e.g., holding an object away from tester after a “give it to me” request; purposely  throwing object across room) |
|  | Response to Social Interaction | Lower | **RSI_Eye Contact** | **S** | Tickle | Child makes eye contact with tester after tester has tickled the child |
|  |  | Lower | **RSI_ACT** | **T** | Tickle | Child vocalizes or bangs the table or reaches to tester after tester has tickled the child |
|  |  | Lower | **RSI_APPEAL** | **U** | Tickle | Child combines RSI_ACT with RSI_EC |
|  |  | Higher | **RSI_Responds To Invitation** | **V** | Response to invitation  (hat, glasses or comb) | Child receives a positive score for each item correctly placed on or toward the adult’s  head in response to invitation “can I play?” (score of 0-3 on this item) |
|  |  | Higher | **RSI_Maintains TurnTaking** | **W** | Turn Taking | Child takes turns with tester throwing the ball or rolling the car (highest number of consecutive turns with tester) |

**Supplementary Table 1. *Coding scheme for non-verbal social-communication behaviors in the Early Social Communication Scales (ESCS).*** *Behaviors are categorized into lower- and higher-level skills across three functional domains: Joint Attention, Behavioral Requests, and Social Interaction. Each domain is further divided into Initiation and Response categories. Task descriptions are adapted from the ESCS manual58.*

**Supplementary analysis 1: The different stages of Model evaluation/validation procedure.**

*K fold cross validation:* The C5.0 model is trained using 10-fold cross-validation (function createFolds of R), dividing the data into 10 subsets to iteratively train and test the model. According to Landis and Koch (Landis & Koch, 1977) a kappa coefficient higher than 0.81 is almost perfect, and given our sample size and to minimize the risk of error, in this study, this strict threshold is chosen to validate decision trees.

*Permutation testing:* Individuals' diagnosis is permuted randomly 100 times, while retaining the same values for the other explanatory variables (ESCS variables) (using the sample function of R). This breaks any real relationship between the explanatory variables and the target variable. The performance of the original model is compared with the average performance of the 100 models created on the permuted data, using T-test to determine whether the observed results are statistically significant or simply due to chance.

*Undersampling:* To compensate for the imbalance between the largest class and the smallest class, undersampling is applied using the ROSE package on R software . Indeed, this disparity could favor the model's performance towards the largest class. A random reduction of the largest class is applied 100 times to avoid chance bias. The average performances of the 100 models created on the undersampled data are compared with the performance of the original model using T-test to judge the influence of the bias induced by class predominance.

*Reference: Landis, J. R., & Koch, G. G. (1977). The measurement of observer agreement for categorical data. biometrics, 159-174.*

**
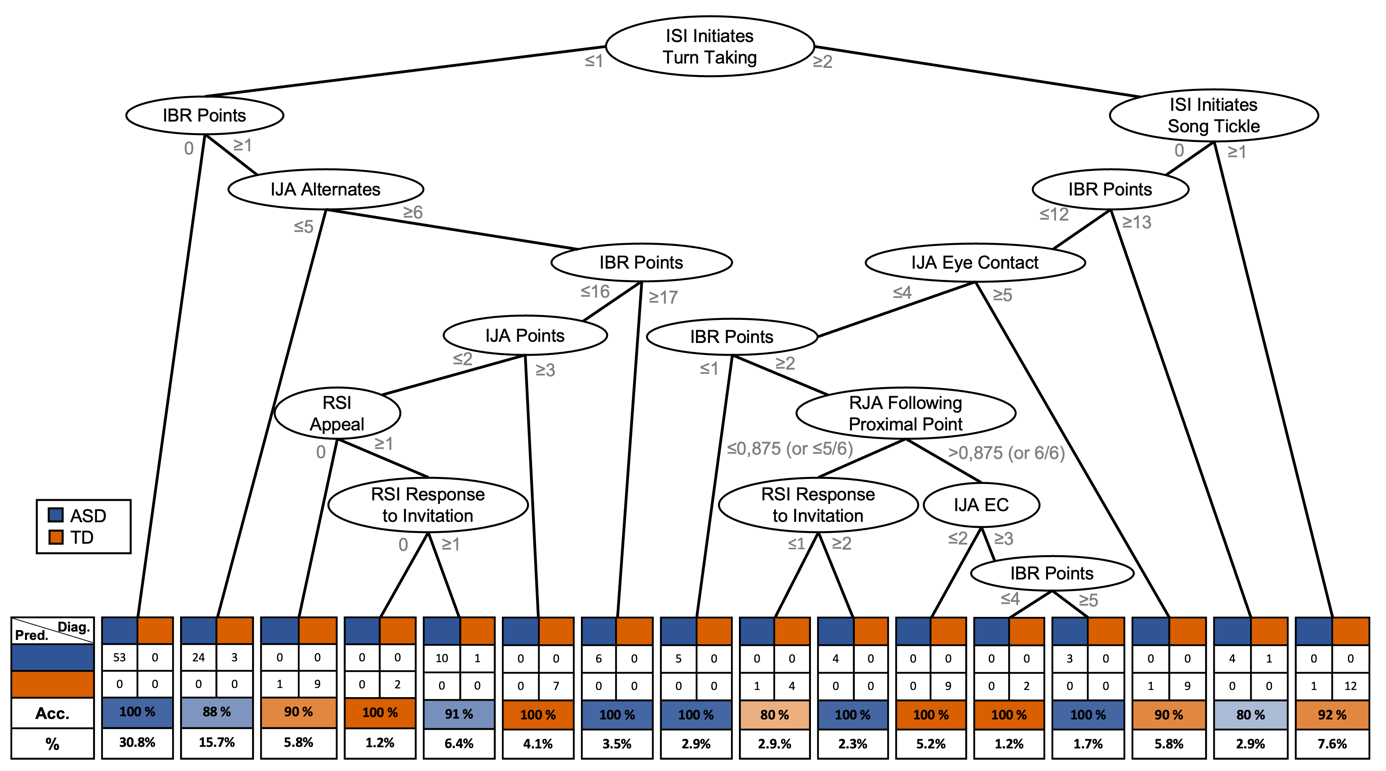
**

**Supplementary Figure 1.** *Diagnosis classification decision tree based on ESCS variables before 3 years old. Thresholds are shown in gray. Diag.: Child’s actual diagnosis. Pred. : Predicted Diagnosis from the Decision tree. Acc.: accuracy of the branch. %: Proportion of children in the total sample represented by this branch.*

**Supplementary analysis 2: Validation of the Autism screening tools**

***K fold Cross Validation of Diagnosis Screening tool***

After 10-Fold cross validation, mean Kappa =0.883, we can then consider that the diagnostic classification model is valid.

***Permutation testing of Diagnosis Screening tool***

To make sure that our diagnostic classification model reflects a real distinction between our ASD and TD groups and not a random one, a permutation testing is applied. The accuracy of the original model (Original model accuracy = 0.948) is statistically better than that of the 100 permuted models (Mean accuracy of the permuted models = 0.774 ; t(2)=2.023, p-value=0.043).

***Undersampling of Diagnosis Screening tool***

To limit class distribution bias, the sample of children with ASD is reduced to equal the TD sample (n=59), 54 ASD children are randomly excluded from the original sample, and this is repeated 100 times to limit chance bias. Accuracy (t(2)=0.882, p-value=0.377) of the 100 trees created on balanced data (mean accuracy on balanced data = 0.940) are not significantly different from original model (original accuracy = 0.948).

| **REFERENCE LETTER** | **TASKS** | **DURATION ESTIMATION (Seconds)** | **DESCRIPTION** |
| --- | --- | --- | --- |
| **A** | Activation of mechanical toy | 20 | Child makes eye contact with tester while manipulating or touching an inactive mechanical toy |
| **B** | Activation of mechanical toy | 20 | Child alternates a look between an active object spectacle and the tester’s eyes |
| **C** | Activation of mechanical toy  + Book | 20 | Before tester has pointed: child points to an active toy OR points to pictures in book OR child points to wall posters |
| **F** | Book | 20 | Tester points to 6 pictures in a book, credit given if child orients head & eyes to picture (calculate as a ratio on the 6 trials) |
| **H** | Activation of mechanical toy | 30 | Child makes eye contact with tester after an object spectacle has ceased OR after the tester has removed an object from the child, to ask him to do it again |
| **K** | Activation of mechanical toy | 20 | Child uses an extended index finger to indicate a desired inactive object or event |
| **O** | Activation of mechanical toy,  Toy in a jar | 20 | Responds to “give it to me”: child gives requested object with or without gesture or shows comprehension with shaking of head or by saying “no” |
| **P** | Turn taking | 30 | Upon receipt of car or ball, child rolls object back to tester this must occur before child has witnessed tester rolling object |
| **Q** | Tickle and song | 30 | Child makes eye contact & runs his fingers across table OR makes tickle gesture OR claps OR sings after the 1st SONG/TICKLE task has been presented |
| **U** | Tickle | 30 | Child combines RSI_ACT with RSI_EC |
| **V** | Response to invitation  (hat, glasses or comb) | 30 | Child receives a positive score for each item correctly placed on or toward the adult’s  head in response to invitation “can I play?” (score of 0-3 on this item) |

**Supplementary Table 2.** Time Estimation. Based on the two longest ESCS videos from the sample of children with ASD (first video: 23 minutes, second video: 25 minutes). Estimation on videos of children with a diagnosis of ASD, who generally show less social behavior in the ESCS assessments, ensure that these are children for whom more opportunities had to be given. Selection of the longest videos ensured that these were children who were more difficult to assess. Overall duration of the ESCS according to the manual = 15 and 25 minutes to assess the 24 total variables included in the evaluation. The screening tool contains a total of 9 variables and the maximum duration is estimated to 12 min.

**Supplementary analysis 3: Decision-tree-based screening tools for the cognitive development of ASD children using membership of the cognitive development clusters as classification variable with three levels (A).**

Decision tree of Cognitive development accuracy achieved 77%.

The final decision tree comprises 7 ESCS variables that are significative in Cognitive development classification.

***Validation of Cognitive Development Classification tree A :***

***K fold Cross Validation of Cognitive Development Classification tree A :***

A K-fold cross validation was carried out, ensuring that the original Cognitive development classification model was reproducible on 10 sub-samples drawn from our population. After 10-Fold cross validation, mean Kappa =0.654 which is not good enough based on the threshold set in this study (0.81), we cannot consider that the Cognitive development classification model is valid.

***Permutation testing of Cognitive Development Classification tree A :***

The average performance metrics of the 100 trees created on permuted data are compared with the performance of the original Cognitive Development classification tree A. The accuracy of the original model (0.766) is not statistically better than that of the permuted models (0.758) (t(2) = 0.071; *p* = 0.94), which means that the original model is not better than models on random data.

**Undersampling of *Cognitive Development Classification tree A :***

Validation by undersampling was not performed because the results of the two previous validation methods invalidated the Cognitive Development Classification tree A.

To improve the performance of this classification tree A for the cognitive development of children with ASD, we propose to classify the two groups of children showing the greatest difference in cognitive development, i.e. including only children who catch up with the cognitive level of the typically developing children at age 5 (Cluster 1), and those children who show very little cognitive development (Cluster 3), and to repeat the same analyses as before.


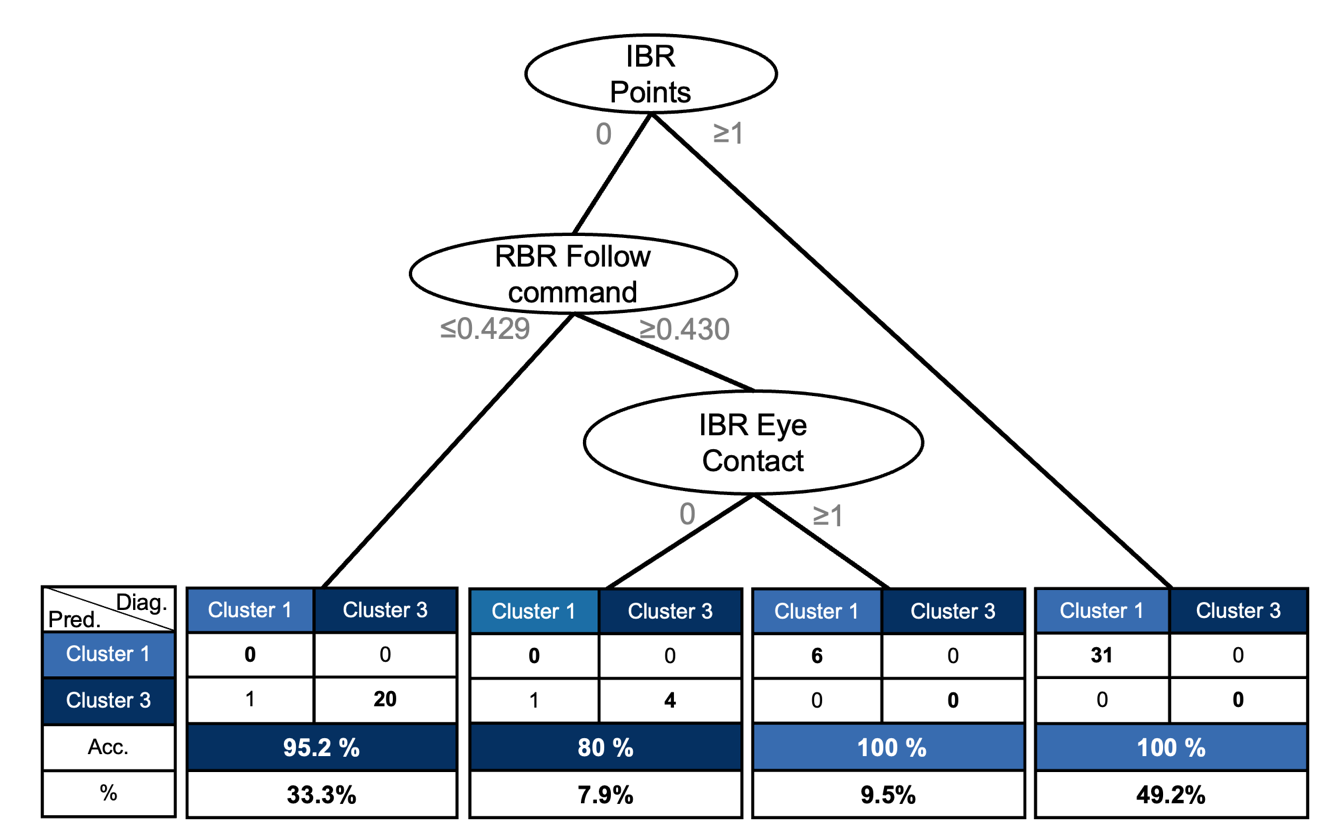


***Supplementary Figure 2.*** *Classification tree of Cognitive development between the two clusters showing the steeper and the weaker developmental trajectories, based on ESCS variables before 3 years old. The thresholds determining each branch of the tree are shown in gray. Diag.: Child’s actual diagnosis Pred.: Predicted Diagnosis from the Decision tree.*

**Supplementary analysis 4:** **Decision-tree-based screening tools for the cognitive development of ASD children using membership of the cognitive development clusters as classification variable with two levels (B)*.***

***K fold Cross Validation of Cognitive Development Classification tree B :***

After 10-Fold cross validation, mean Kappa =0, 933, we can then consider that the diagnostic classification model is valid.

***Permutation testing of Cognitive Development Classification tree B :***

To make sure that our diagnostic classification model reflects a real distinction between our ASD and TD groups and not a random one, a permutation testing is applied. The accuracy of the original model (original accuracy = 0.968) is statistically better than that of the 100 permuted models (Mean accuracy of the permuted models = 0.710 ; SD = 0.081 ; t(2)=3.172, p-value=0.002)

**Undersampling of *Cognitive Development Classification tree B :***

To limit class distribution bias, the sample of children with ASD is reduced to equal the TD sample (n=59), 54 ASD children are randomly excluded from the original sample, and this is repeated 100 times to limit chance bias. Accuracy (t(2)=-0.344, p-value=0.73) of the 100 trees created on balanced data (mean accuracy on balanced data = 0.972 ; SD=0.013) are not significantly different from the original model (original accuracy = 0.968).

**Supplementary analysis 5: Post-hoc classification of Cluster 2 children using the binary decision tree model.**

The aim of this analysis is to explore whether the decision tree model trained to distinguish between Cluster 1 and Cluster 3 children could meaningfully classify children from the intermediate Cluster 2 group, and whether these assigned labels corresponded to clinically distinct developmental profiles.

To do so, we applied the decision tree constructed to predict long-term cognitive outcomes based on early socio-communicative behaviors (trained on Cluster 1 and Cluster 3 only) to children from Cluster 2 (n = 48). The resulting predicted labels assigned each child to either a “Cluster 1-like” or “Cluster 3-like” group.

We then compared the following developmental variables between the two subgroups:

- Age at first visit
- Cognitive level at first visit (age-equivalent score)
- Cognitive level at last visit (age-equivalent score)
- Slope of cognitive growth (regression slope between first and last cognitive evaluation)

Statistical comparisons were performed using independent samples t-tests, with Levene’s test for equality of variances applied to guide interpretation.

| Variable | Cluster 1-like (n=35) | Cluster 3-like (n=13) | Mean difference | p-value |
| --- | --- | --- | --- | --- |
| Age at first visit (months) | M=29.53 (SD=3.78) | M=27.73 (SD=3.65) | 1.79 | 0.149 |
| Cognitive Level at first visit (age equivalent) | M=21.43 (SD=5.23) | M=16.45 (SD=3.79) | 4.97 | **0.003** |
| Cognitive Level at last visit (age equivalent) | M = 42.39 (SD=6.88) | M=36.84 (SD=10.21) | 5.55 | **0.035** |
| Slope of cognitive growth | M=0.92 (SD=0.17) | M=0.95 (SD=0.18) | -0.028 | 0.612 |

**Supplementary table 3**. Comparison of “Cluster 1-like” and “Cluster 3-like” sub-groups of Cluster 2 children. Significant differences are highlighted in bold.

These results suggest that Cluster 2 contains at least two distinct subgroups: one with relatively high cognitive functioning from baseline and sustained gains, and another with lower functioning and more limited developmental progress. While these groups showed similar rates of developmental growth, they differed significantly in absolute cognitive level. The fact that the decision tree could meaningfully classify Cluster 2 children, despite being trained only on Clusters 1 and 3, supports the discriminative value of the three early socio-communicative behaviors highlighted by the tree.
