# Supplementary material for: A Brief Observation to Screen Autism in Toddlers and Predict Developmental Trajectory
Source: J Pediatr Clin Pract. 2025 Sep 1;18:200176. doi: 10.1016/j.jpedcp.2025.200176 (PMC12766091; doi:10.1016/j.jpedcp.2025.200176)
Supplement: Data Statement [file mmc2.docx]

Data Statement

The data supporting the findings of this study are available upon reasonable request from the corresponding author. Due to ethical and privacy considerations, the dataset cannot be made publicly available, as it contains sensitive information about child participants.
